# Histiocytic necrotizing lymphadenitis with central nervous system involvement in children: An analysis of clinical features

**DOI:** 10.12669/pjms.42.9.16518

**Published:** 2026-09

**Authors:** Xiaoyuan Wu, Fang Guo, Yanhong Jia, Wenjun Luan, Chunxia Yang

**Affiliations:** 1 Xiaoyuan Wu, Department of Infectious Diseases, Hebei Children’s Hospital, Shijiazhuang 050000, Hebei, China; 2 Fang Guo, Department of Infectious Diseases, Hebei Children’s Hospital, Shijiazhuang 050000, Hebei, China; 3 Yanhong Jia, Department of Infectious Diseases, Hebei Children’s Hospital, Shijiazhuang 050000, Hebei, China; 4 Wenjun Luan, Department of Infectious Diseases, Hebei Children’s Hospital, Shijiazhuang 050000, Hebei, China; 5 Chunxia Yang, Department of Infectious Diseases, Hebei Children’s Hospital, Shijiazhuang 050000, Hebei, China

**Keywords:** Central nervous system, Children, Histiocytic necrotizing lymphadenitis

## Abstract

**Objectives:**

To summarize the clinical features of histiocytic necrotizing lymphadenitis (HNL) with central nervous system (CNS) involvement in children.

**Methodology:**

A retrospective analysis was conducted on the clinical data of HNL-CNS children diagnosed and treated in Hebei Children’s Hospital from January 2015 to January 2025.

**Results:**

A total of 22 HNL-CNS children were enrolled (17 boys, five girls; age 9.2 ± 3.5 years). Fever and lymphadenopathy occurred in all cases. Neurological manifestations included headache/vomiting (77.3%), convulsions (27.3%), and consciousness disorders (18.2%). Eight children presented with neurological symptoms before lymphadenopathy. Peripheral blood leukopenia was found in 68.2%. Cerebrospinal fluid (CSF) showed elevated pressure (81.8%), elevated white blood cell count (86.4%, mainly monocytes), and elevated protein (50.0%). Cranial MRI abnormalities were observed in 68.2%, predominantly in frontal/occipital lobes, with leptomeningeal enhancement in 13.6%. Anti-Mycoplasma pneumoniae antibody titers ≥1:160 were found in 50.0%. Antiviral therapy with acyclovir was given to 19 (86.4%) children, antibiotics were used in 17 (77.3%) children, intravenous corticosteroid (CS) therapy was provided to 19 (86.4%) children, and Cs combined with intravenous immunoglobulin (IVIG) was adopted in 8 (36.4%). Two out of three children who did not use Cs experienced recurrences.

**Conclusion:**

HNL-CNS occurs predominantly in school-age boys. Neurological symptoms may precede lymphadenopathy. CSF findings are characterized by elevated pressure and mononuclear pleocytosis. MRI lesions commonly involve frontal and occipital lobes. Corticosteroid therapy yields favorable clinical outcomes and a low recurrence risk.

## INTRODUCTION

Histiocytic necrotizing lymphadenitis (HNL) is a rare benign histiocytic proliferative disorder of unknown etiology,^1^ which is often characterized by fever, lymph node enlargement, rashes and leukopenia, and can involve multiple organs such as the liver, kidney, blood vessels, skin and bone marrow^2^ and even the central nervous system (CNS) in a few patients, causing encephalitis, aseptic meningitis,^3^ subdural effusion^4^ and cerebellar ataxia. HNL has been reported worldwide, with higher prevalence in Asian populations, particularly in Japan, South Korea, and China.

Compared with adults, children with HNL show striking histological and immunopathological similarities with systemic lupus erythematosu s (SLE) clinically, including the presence of apoptotic karyorrhectic debris, plasmacytoid dendritic cell infiltration, and activation of the interferon-alpha pathway. Longitudinal studies have documented that a subset of HNL patients may progress to SLE months to years after initial diagnosis, suggesting HNL may represent a prodromal or specific manifestation of SLE in susceptible individuals.

Only a few cases of HNL-CNS have so far been reported without definite clinical features. Some children present with meningitis as the initial manifestation,^5^ followed by lymph node enlargement, which is prone to misdiagnosis and missed diagnosis in clinical practice. The diagnosis of HNL-CNS is particularly challenging because neurological symptoms may precede peripheral lymphadenopathy, leading to delays in appropriate treatment and unnecessary use of anti-infective agents. Given the substantial clinical uncertainty, potential for severe neurological sequelae, and high risk of misdiagnosis, a comprehensive analysis of HNL-CNS in children is urgently needed. We here retrospectively summarize the clinical data of HNL-CNS children treated in Hebei Children’s Hospital, so as to provide assistance for rational diagnosis and treatment.

## METHODOLOGY

A retrospective analysis was conducted on the clinical data of HNL-CNS children diagnosed in Hebei Children’s Hospital from January 2015 to January 2025.

### Ethical approval:

The study was approved by the Institutional Ethics Committee of Hebei Children’s Hospital (No.2024147; Date: June 6, 2024). Written informed consent was obtained from the parents or legal guardians of all participants.

### Inclusion criteria:

- An age between one and 18 years.
- HNL confirmed by lymph node biopsy.
- Presence of CNS damage (including aseptic meningitis, intracranial hypertension, status epilepticus, subdural effusion and cerebellar ataxia).

### Exclusion criteria:

- Congenital immunodeficiency and genetic metabolic diseases.
- CNS diseases induced by traumas, poisonings, etc.

### Clinical data collection:

The clinical data including gender, age, length of hospital stay, mode of onset, clinical manifestations, treatment, complications and outcomes were collected.

### Auxiliary examinations: Laboratory tests:

Venous blood: White blood cell (WBC) count, minimum WBC count, hemoglobin (Hb), platelet (PLT) count, C-reactive protein (CRP), erythrocyte sedimentation rate (ESR), procalcitonin (PCT), blood culture, blood biochemistry, ferritin (FER), autoantibody spectrum, antinuclear antibodies, total lymphocyte immunoassay, immunoglobulin (Ig) quantification, anti-streptolysin O (ASO) antibody, anti-Epstein-Barr (EB) virus antibody and PCR, anti-Mycoplasma pneumoniae (MP) antibody, and T cell spot test (T-SPOT) were determined upon admission.

### Cerebrospinal fluid (CSF):

All the children underwent lumbar puncture within 24 hours after the onset of neurological symptoms. CSF was collected for pressure measurement, and sent for routine test, biochemical test, smearing, ink and acid-fast staining, herpes simplex virus I/II PCR, MP PCR, and EB virus PCR.

### Bone marrow:

The bone marrow was smeared and cultured. Imaging examinations: lymph node ultrasound, electroencephalography (EEG), and cranial magnetic resonance imaging (MRI) were carried out.

### Statistical analysis:

The data were analyzed using SPSS 23.0 statistical software. Non-normally distributed measurement data were expressed as median (M) (P25~P75) or median (full range), and compared between the two groups with the U test. Comparisons of the measurement data with a normal distribution were performed by the t test. Enumeration data were described by n (percentage), and their intergroup comparisons were conducted using the χ^2^ test or Fisher’s exact probability test. P < 0.05 was considered as statistically significant.

## RESULTS

In this study, a total of 465 HNL children were collected, and finally 22 HNL-CNS children were enrolled, including 17 boys and five girls, with an onset age of 9.2 ± 3.5 years. There were four children under six years old, all of whom were boys, and 16 children aged 6-12 years, including 12 boys and four girls. The median length of hospital stay was 21.0 (17.0, 25.0) d. Among them, eight children initially presented with neurological manifestations, three with fever accompanied by lymph node swelling and pain, and 11 with fever alone. During follow-up, recurrences occurred in four children, with HNL-CNS in one child and HNL alone in three children, and an interval to initial onset of 21 (3.3, 44.0) months. One child experienced recurrences twice, with an interval to initial onset of four and six years, respectively. Sequelae were identified in two children, both of whom exhibited epilepsies without deaths.

Fever and lymph node enlargement occurred in all 22 children, of which 14 (63.6%) suffered from aseptic meningitis, 7 (31.8%) from encephalitis and one (4.5%) from disseminated encephalomyelitis. The most common neurological manifestations were headache and vomiting (n = 17, 77.3%), followed by convulsions (n = 6, 27.3%), disorders of consciousness (n = 4, 18.2%) and status epilepticus (n = 2). Other clinical manifestations included rashes (n = 11, 50.0%), chills (n = 10, 45.5%), oral ulcers (n = 5, 22.7%), hepatomegaly or splenomegaly (n = 5, 22.7%), and joint swelling and pain (n = 4, 18.2%). Two (9.1%) children were complicated with respiratory failure and treated with mechanical ventilation, and one (4.5%) child was concurrent with macrophage activation.

At HNL-CNS diagnosis, reduced WBC count was detected in 15 (68.2%) children, among which 10 (45.5%) had a decrease in WBC count upon admission, decreased Hb in 7 (31.8%) children, increased CRP in 12 (54.5%) children, elevated ESR in 17 (77.3%) children, serum FER > 500 mg/L in 2 (9.1%) children, and increased alanine aminotransferase (ALT) in 12 (54.5%) children, with the median liver enzyme of 238.5 (150.3, 440.0). There were 11 (50.0%) children with an anti-MP antibody titer ≥ 1 : 160, 9 (40.9%) with elevated ASO, and 2 (9.1%) with EB viral DNA-positive. Autoantibodies were positive in 6 (27.3%) children, of which four were anti-Ro52KD antibody-positive, one was anti-SS-A antibody-positive and 1 was anti-JO-1 antibody-positive. Two (9.1%) children exhibited antinuclear antibody-positive, with low titers. Anti-IgG antibody elevated in 2 (9.1%) children, and anti-IgM antibody increased in 5 (22.7%) children. Twenty-one (95.5%) children underwent lymphocyte immunoassay, revealing that CD4+ T-lymphocyte levels reduced in 10 (45.5%) children, while CD8+ T-lymphocyte levels increased in 8 (36.4%) children. According to bone marrow puncture, hematological malignancies were excluded. CSF examination showed that 18 (81.8%) children had elevated CSF pressure to varying degrees, with three children presenting simple CSF pressure elevation without pleocytosis. CSF WBC count elevation (> 5 × 10^6^/L) was observed in 19 (86.4%) children, with a median of 58.0 (31.0, 201.0) and a range of 20-529 × 10^6^/L, with the predominant cell type being monocytes. Eleven (50.0%) children exhibited CSF protein elevation (> 400 mg/L), with a median of 0.8 (0.44, 1.66) and a range of 0.42-2.45 g/L. Glucose and chloride levels in CSF were mostly within the normal ranges. No abnormalities were found in CSF bacterial and fungal cultures among the 22 children.

All the 22 children received cranial MRI, which suggested intracranial lesions in 15 (68.2%) children, involving the cerebral cortex, basal ganglia and globus pallidus, and displayed abnormal long T2 signals and high FLAIR signals. In 8 (36.4%) children, the lesions were located in the cerebral hemisphere, mostly in the frontal and occipital lobes, and presented varying volumes and multiple punctate or patchy distribution, partially accompanied by cortical swelling, without bleeding or necrosis (Fig.1a). Four (18.2%) children exhibited widening of cerebral sulci and extracerebral space, and the rest 3 (13.6%) had leptomeningeal enhancement (Fig.1b).

**Fig.1:**
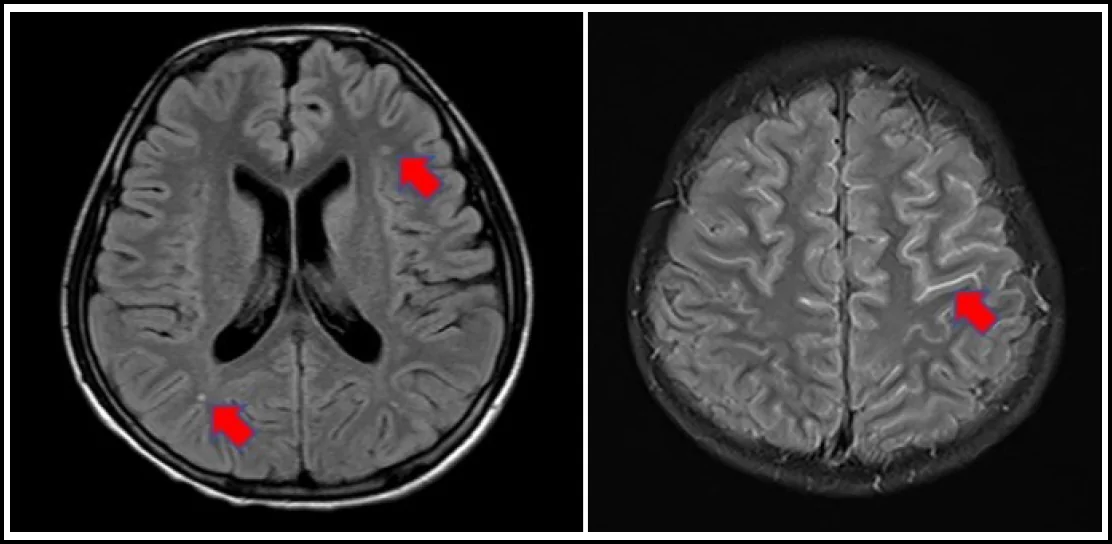
(left: a, right: b): Cranial MR images of children with HNL-CNS.a: Punctate or patchy abnormal signals in the frontal and occipital lobes, and lesions of varying volumes; b: Leptomeningeal enhancement.

All the children presented destruction of the lymph node structure, with only a small amount of residual lymphatic tissue and follicles at the edges, large areas of necrosis and nuclear debris, and histiocyte proliferation. Immunohistochemical staining suggested CD3 (+) T cells, CD20 (+), CD21 (dendritic cells +), CD68 (+), and in situ hybridization: EBER (-) (Fig.2).

**Fig.2:**
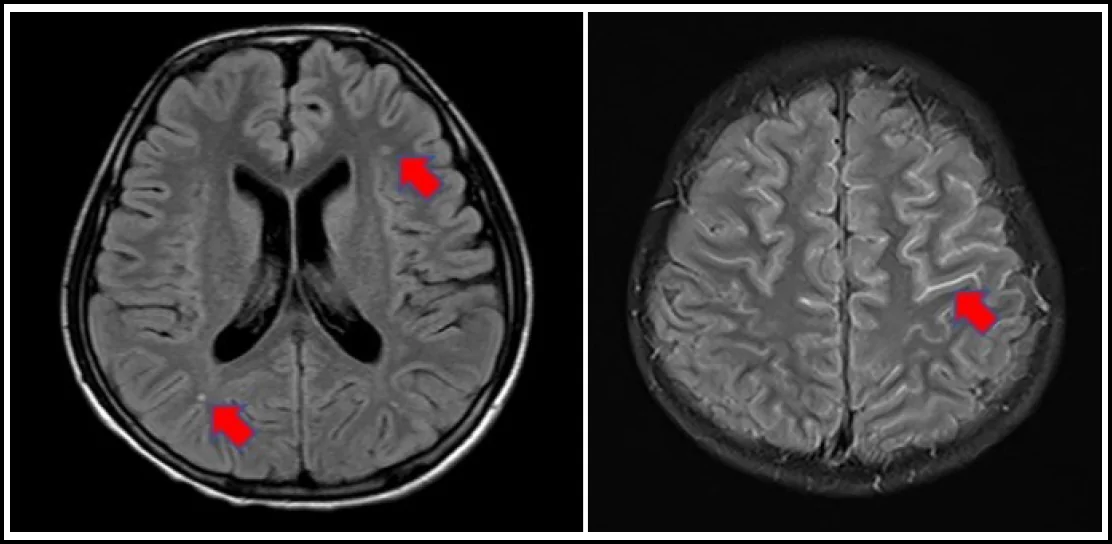
Lymph node biopsy results of children with HNL-CNS (left: HE staining, right: CD68+).

Antiviral therapy with acyclovir was given to 19 (86.4%) children and discontinued after confirmation by lymph node biopsy, with the course of treatment ranging from one to two weeks. Seventeen (77.3%) children were treated with antibiotics, including third-generation cephalosporin in 15 children and azitromycin in six children. Six (27.3%) children were treated with combined use of antibiotics. Intravenous corticosteroid (CS) therapy was given to 19 (86.4%) children, of which 8 (36.4%) were treated with CSs combined with human intravenous immunoglobulin (IVIG). Compared with CS therapy alone, the combined use showed no advantages in relieving symptoms and reducing recurrences, as seen in Table-I. After discharge, low-dose CSs were orally administered for maintenance treatment. Two out of three children who did not use CSs experienced recurrences, and one of them had a HNL-CNS recurrence one month after the onset.

**Table-I:** Comparison of clinical efficacy between HNL-CNS children with CS alone and CS + IVIG.

	CS alone (n = 11)	CS + IVIG (n = 8)	Statistic	P
Fever relief (d)	3.0(2.0, 5.0)	3.5(2.3, 4.8)	0.073	0.717
Relief of lymph node swelling and pain (d)	7.0(6.0, 9.0)	8.0(7.0, 11.3)	0.273	0.310
Normal CSF (d)	11.5±3.6	11.1±5.5	0.202	0.842
Recurrence [n (%)]	1(9.1)	1(12.5)	--	1.000

## DISCUSSION

In this retrospective study of 22 pediatric patients with HNL-CNS, we found that the condition predominantly affected school-age boys, with aseptic meningitis being the most common CNS manifestation. Notably, 36.4% of patients presented with neurological symptoms prior to the appearance of lymphadenopathy, highlighting a diagnostic challenge. CSF analysis consistently revealed elevated pressure and mononuclear-predominant pleocytosis, while cranial MRI frequently demonstrated frontal and occipital lobe lesions. Corticosteroid therapy was associated with rapid clinical improvement and a low recurrence rate, whereas patients who did not receive corticosteroids had a significantly higher recurrence rate.

HNL occurs frequently in Asian countries including South Korea and Japan,^6^ with age-dependent susceptibility and histological characteristics that overlap with systemic lupus erythematosus (SLE), and it can develop into SLE. The histopathological overlap between HNL and SLE includes the presence of abundant apoptotic karyorrhectic debris, plasmacytoid dendritic cell infiltration, and elevated type-I interferon pathway activity. Several longitudinal studies have documented cases of HNL progressing to SLE months to years after initial diagnosis, suggesting that HNL may be an early or prodromal manifestation of SLE in a subset of patients. It has been shown that children mostly present with fever, lymph node swelling and pain, and rashes, and 5.9% of HNL children develop immune-mediated rheumatic diseases such as SLE and Sjogren’s syndrome. However, HNL-CNS is rare in clinical practice.^7^ In the past 10 years, HNL-CNS patients in our hospital only accounted for 4.7% (22/465) of the total number of HNL patients. In the present study, HNL-CNS children including 17 boys and five girls were enrolled, and it was found that HNL-CNS in our cohort was more common in boys at school age. Additionally, boys with HNL were more prone to neurological damage compared with girls in this study. However, given the small sample size, this gender and age predominance should be interpreted with caution and requires validation in larger multicenter studies. No immune-mediated rheumatic diseases such as SLE occurred during the follow-up, so it is speculated that HNL-CNS may be a special entity of HNL.

HNL-CNS presents with complex and diverse manifestations, including meningitis, encephalitis, subdural effusion, optic neuritis, cerebellar ataxia and hemiplegic symptoms. This study found that aseptic meningitis was the most common CNS complication of HNL, which is consistent with previous research results.^8^ The most common symptom in patients with aseptic meningitis is headache,^9^ followed by neck discomforts, disorders of consciousness, and convulsions. In our study, all the 14 children with meningitis suffered from headache and vomiting, which may be related to vasogenic cerebral edema caused by increased permeability of cerebral capillary endothelial cells, as well as to intracranial pressure elevation due to inflammation-induced damage and adhesion of the meningeal surface, and disturbance in CSF absorption and reflux.

Song Y et al.^9^ reviewed all 41 case reports on HNL-CNS in the PubMed database during 1979-2020 and found that up to 86% of cases had elevated CSF WBC counts, nine cases had elevated CSF pressure, but 21 cases had no relevant mentions. Our study revealed elevated CSF WBC count in 19 (86.4%) children, elevated CSF pressure in 18 (81.8%) children, increased CSF proteins in 11 (50.0%) children, and normal glucose and chloride levels in CSF, which leads to a difficulty in distinguishing from viral encephalitis and tuberculous meningitis. The consistency between our findings and those of previous reports suggests that CSF pleocytosis with mononuclear predominance, elevated opening pressure, and normal glucose are characteristic features of HNL-CNS. However, the absence of hypoglycorrhachia and the relatively mild protein elevation may help differentiate HNL-CNS from bacterial or tuberculous meningitis. In addition, eight children in this study presented with meningitis-like symptoms prior to lymph node enlargement, which posed a great challenge to the diagnosis of HNL-CNS and was likely to cause the abuse of anti-infective drugs. Therefore, for aseptic meningitis with unknown etiology in which it is difficult to distinguish CSF results from relevant CNS infections, especially accompanied by rashes and a decline in peripheral blood WBC count, lymph node lesions should be carefully examined, and if necessary, lymph node biopsy should be performed to determine the presence of HNL-CNS.

It has been reported that the MRI lesions of HNL-CNS patients are mainly in the cerebellum, brainstem, temporal and occipital lobes, with observable irregular meningeal enhancement and subdural exudation.^10^ In our study, cranial MRI suggested that the lesions of 36.4% children were located in the frontal and occipital lobes, 18.2% children exhibited widening of cerebral sulci and extracerebral space, and 13.6% had leptomeningeal enhancement. The predominant involvement of the frontal and occipital lobes in our cohort differs somewhat from previous reports, which may be related to differences in disease severity, timing of MRI acquisition, or patient age distribution. Notably, children with leptomeningeal enhancement had significantly higher CSF WBC counts, suggesting that meningeal inflammation is closely linked to imaging abnormalities. Moreover, these children had significantly elevated CSF WBC counts, and the increased mononuclear cells infiltrated into the meninges, which caused obstruction and stasis of the capillary network, and an increase in endothelial cell pressure and permeability, thus resulting in widening of the extracerebral space or subdural effusion.

The etiology and pathogenesis of HNL are still unclear. According to its self-limiting disease course and effective Cs therapy, most scholars believe that HNL is related to viral infection and its mediated autoimmune responses.^11^,^12^ MP is currently one of the important pathogens causing infections in children. In addition to respiratory infections, it can also damage the nervous system through direct invasion into the brain, immune-mediated inflammatory responses, neurotoxin-induced damage or thromboembolism, causing encephalitis, meningoencephalitis, cerebellitis, cerebral infarction and acute disseminated encephalomyelitis.^13^,^14^ At present, there have been a few reports on MP infection-induced HNL,^15^,^16^ but its mechanism is still unclear. In the present study, 11 (50%) children exhibited elevated anti-MP antibody titers(≥1:160), suggesting a possible association between MP exposure and HNL-CNS. However, it is important to note that a single elevated antibody titer does not confirm acute MP infection, as paired acute and convalescent sera showing a fourfold rise in titers or MP PCR positivity are required for definitive diagnosis. Since such confirmation was not obtained in this retrospective study, we acknowledge this as a limitation and interpret the association as suggestive rather than causal. The severity of MP-induced lesion is correlated with immune responses, but not with MP load:^17^

MP directly invades and destroys epithelial cells. With the participation of miRNA-492, it binds to antigenic components to form immune complexes, thereby activating the complement system and lymphocytes, inhibiting immune function, and disrupting immune regulation.^18^

MP stimulates macrophages to release inflammatory cytokines and chemokines through Toll-like receptors, leading to immune dysfunction.^19^

Under the action of surface-immobilized polyclonal immunoglobulin-binding proteins, MP resists host immunoglobulins, thus disrupting the function of the immune system.^20^ Furthermore, the massive aggregation of T lymphocytes and CD68 (+) macrophages displayed in lymph node and dural biopsy for HNL-CNS^10^ is the key to triggering abnormal immune responses. Based on these observations and our findings, we speculate that MP infection and its mediated immune responses may be associated with the pathogenesis of HNL-CNS, though a causal relationship cannot be established from this study.

As a self-limiting disease, HNL has no relevant therapeutic standard or consensus so far. A follow-up study in China has shown that fever and lymph node enlargement in 48.3% children self-heal after symptomatic treatment, and 20.3% children experience recurrences.^7^ In our study, 19 (86.4%) children received Cs therapy, with two children experiencing recurrences, whereas those who did not use Cs had a significantly higher recurrence rate. Additionally, lymph node swelling and pain, and intracranial hypertension in children treated with Cs were relieved rapidly, which can reduce the application time of dehydrating agents such as mannitol. Six children were treated with IVIG in combination, and compared to Cs therapy alone, the combination of Cs and IVIG showed no advantage in terms of efficacy. Meanwhile, significant abuse of anti-infective drugs was found during the diagnosis and treatment of HNL-CNS in children. In this study, 19 (86.4%) children were given antiviral drugs, 17 (77.3%) children were given antibacterial drugs, and 6 (27.3%) children were treated with combined use of antibiotics, all of which were discontinued after lymph node biopsy. The reasons may be as follows:

- The early manifestations of HNL-CNS are nonspecific. Fever, rashes and organ involvement can be the manifestations of infectious diseases.
- Clinicians lack experience and fail to pay timely attention to lymph node enlargement in children.
- Early CSF results of HNL-CNS are difficult to distinguish from CNS infections.

### Strengths:

The primary strength of this study lies in its detailed characterization of a relatively large pediatric HNL-CNS cohort over a decade, offering novel insights into its natural history, diagnostic biomarkers (CSF/MRI), and treatment response. These findings fill a critical gap in the pediatric neurology literature by establishing a preliminary framework for recognizing and managing this rare complication of HNL.

### Limitations:

Limitations include the retrospective design, single-center setting, modest sample size, and lack of definitive confirmation for suspected MP infections.

## CONCLUSIONS

In this cohort, pediatric HNL-CNS occurred predominantly in school-age boys. Neurological symptoms such as headache, vomiting and convulsions may appear prior to lymph node enlargement. CSF findings are characterized by elevated pressure, mononuclear-predominant pleocytosis, and variable protein elevation. MRI lesions commonly involve the frontal and occipital lobes, with possible meningeal enhancement. Corticosteroid immunotherapy is associated with favorable outcomes and a low recurrence rate. Clinicians should maintain a high index of suspicion for HNL-CNS in children presenting with aseptic meningitis of unknown etiology, particularly when accompanied by rash, leukopenia, or cervical lymphadenopathy.

### Recommendations:

Future prospective, multicenter registries incorporating standardized immunological profiling and pathogen detection are warranted to validate these findings and elucidate underlying mechanisms.

### Authors’ Contributions:

**XW** and **FG:** Carried out the studies, data collection, and drafted the manuscript, and are responsible and accountable for the accuracy or integrity of the work;

**YJ:** Performed the statistical analysis and participated in its design;

**WL** and **CY:** Prepared the manuscript and Figures 1-2.

All authors have read and approved the final manuscript.
